# On the Organizing of the American Medical Association

**Published:** 1852-03

**Authors:** Samuel Jackson

**Affiliations:** President; Formerly of Northumberland


MEDICAL EXAMINER,

AND

RECORD OF MEDICAL SCIENCE.

NEW SERIES.—NO. LXXXVII.—MARCH, 1852.

### ORIGINAL COMMUNICATIONS.

*On the Organizing of the American Medical Association. Read before the Philadelphia County Medical Society, Feb.* 1852. By the President, SAMUEL JACKSON, M. D., formerly of Northumberland. [Printed by order of the Society.]

NOTE.—The subject of the following discourse was not discussed by the Society owing probably to the lateness of the hour; but it was ordered to be published and largely disseminated *nem. dissent.* the meeting being very full.

Americans have always considered that government has its just foundation, beginning, and *primum mobile* in the will of the people; and ample experience, both in the general and state governments, has shown that they have not been mistaken. Upon the same principle, every church in the United States, except one, has established its government; and here too, success has demonstrated that wisdom presided at their councils and directed the religious republics. Even the Church of England, when brought to America, suddenly and spontaneously assumed a republican form. It might be said in a metaphor, that transplanted to our soil, it sprang up a beautiful tree of liberty, bearing flowers and fruits delighting to the eyes and pleasing to the taste of a simple republican people. In England, all power in the church proceeds from the Queen. She nominates the Bishops, they appoint the Rectors, these appoint their Vicars, the people of the parishes being obliged to receive whatever is imposed upon them in the shape of clergymen. In America, on the contrary, the parishoners elect their clergymen, a convention of the clergy and laity elect their Bishop. Thus all power in this church proceeds from the people, as it ought ever to do, when the people are qualified to govern themselves.

In accordance with these illustrious examples in Church and State, the physicians of America ought surely to be governed: that is, by an equal and just representation from their own body. This we cannot doubt is their will and their will ought to be respected and obeyed. The American Medical Association is not a republican institution—it is aristocratical both in its origin and in its continuance. Of this however we do not complain. Its origin was anomalous, but there was perhaps no other way in which a large body of respectable men could be so quickly got together. In all cases of difficulties, dangers, or pressing necessities, that cannot be met or subserved in the regular course of action, some commanding spirits may assume a temporary authority. Thus when the stamp-act required the immediate action of the American people, the Assembly of Massachusetts Bay took measures to have a Congress called from all the Colonies to meet at New York, and ten Colonies obeyed the summons. So when the state of our Profession in America seemed to require that support and protection which the laws denied, the Medical Society of New York summoned a convention which has resolved itself into the American Medical Association.

This has now become nearly *functus officio*, it has acted its part well, it has done its duty, and it is now time for it to prepare for dissolution, in order to be re-constituted in a republican form. It assumed power and has used it well; but now when the continuance of this power is no longer necessary, it cannot be patiently yielded; let the Association then lay it down and grant to the people, or let them assume, their just representative government.

To the continuance of the present system much longer, some valid objections have been made.

The Profession is very unequally represented. The Constitution says—"The Delegates shall receive their appointment from permanently organized medical societies, medical colleges, hospitals, lunatic asylums, and other permanently organized medical institutions of good standing."

Now it happens that in cities, a physician who is a member of several institutions, is elected by them all, and he goes to the Association as a Bashaw with many tails. Sometimes he rejects his supernumerary honors and calls himself a delegate from some favorite institution only. Colleges furnished with five or seven professors, send two of their members, and hence it happens, that the collective colleges in the United States have an over proportion of representation. This has created dissatisfaction, and to a degree apparently over-proportioned to the cause; it resembles too much the election of members of Parliament from the rotten boroughs of England. So when an hospital or any other institution is supplied with only one physician, this one may go every year, to represent himself.

All permanently organized medical institutions of good standing are permitted to send delegates. Now who is to determine whether certain institutions are *permanently organized*, and also, whether they are *of good standing?* "Oh! the Association will reject at its meeting all that do not come within the letter, provided an accusation is brought." Indeed! but this would be a very troublesome procedure and extremely invidious, rendering the accuser almost hateful. It occurred in Philadelphia, in Charleston, and it was on the very verge of giving infinite trouble at Boston.

Again—two or three physicians who would not be received into any Association *of good standing*, may suddenly call themselves a *Society permanently organized*, and then send a delegation. When you hear this new Society announced at the Association, you are ready to cry out like Dido—who is this stranger—*Quis novus hic nostris successit sedibus hospes?* There does not appear any reason whatever that we shall not see a delegation to Richmond from the female medical college. Nay, it is highly probable that next May will exhibit such a monstrous medical phenomenon as will leave nothing worse for posterity to gaze upon.*

It has been strongly objected to the present constitution, that a very small proportion of the physicians of the United States can ever become members. The Association has now been continued through five annual meetings, and it does not number 920 both living and dead. Many of these have attended every meeting, and not a few will continue to attend every year as long as they live; partly because they are connected with colleges, hospitals, and other associations, each consisting of a small number of members. Some plan, it has been thought, ought therefore, to be adopted for rendering the institution more catholic, by which every respectable physician in the Union may attain membership.

One great object of the Association is to render the profession more respectable by improving its individual members; for in every great body of people, as clergymen, lawyers, and physicians, however high their pretensions, there will always be found some who are hardly worthy of fellowship. Now if there are any means of tempting these people to improve themselves, it consists in firing their ambition. Hold out to them the hopes of a fellowship in the great Association, and they will soon prove themselves worthy thereof. When they have the watchful eyes of the County Society upon them, they will improve daily in all good properties and qualities.

Not a few members of the Association hold themselves forth as such in the title pages of their various publications; now if this membership exalts even authors and teachers in their own estimation, how greatly will inferior men be delighted to have it known in their villages, that they too have been favored with this enviable distinction! Thus in a village, where every one knows the history and present state of his neighbor and where no dereliction can pass unnoticed, a physician will not rest night or day till he render himself worthy of a membership in the County Society; for admission to this, according to the plan we are about to propose, makes him at once a member of the great Association of American Physicians.

* Nil erit ulterius quod nostris moribus addat
Posteritas—— *Juv. Sat.* i.

The admission of members *by invitation*, is thought highly objectionable. *Delegates* are sent to transact the business of the American physicians; to them and to them only it ought to be confided. Suppose Congress or the State Legislatures had permission to invite some favorites into their body, with the privilege of declaiming and voting, what think you would be the result? Moreover the inviting of these members is an invidious thing. Some will be admitted and some rejected, sometimes through ignorance and sometimes through caprice, or something worse. I have seen the most worthy rejected and the most unworthy admitted.

The privilege granted to what are called *permanent members*, of attending all subsequent meetings and partaking in the debates, is a monstrous anomaly and an outrage on common sense. Grant to ex-members of Congress the same privilege, and when think you would their debates find an end? Moreover, a man who is a delegate this year, may soon be turned out of his society for misconduct. How would you feel in meeting him at the Association as a permanent member?

The organization to be now proposed is this:

1. Let the Association be composed of Delegates from County Societies only.

2. Let every man receive, as soon as he is elected into his County Society, a diploma, testifying that he is a member of said County Society, of the State Society, and of the American Medical Association. He is then one of the great body of the brethren in the United States; he is ready to be elected a delegate whenever his fellows see fit to elect him; he is ready to give his vote in the election of others; he is now a member of the great medical republic of his country. He therefore values his diploma from the County Society more than that from the University; for the latter only testifies that he is a Doctor of Medicine, the former makes known that in addition to this, he is now a respectable citizen, a practitioner in good repute, and a member of the American Medical Association. Tis true, he may never be made a delegate, time and chance happeneth to all men; he may not even desire this honor, but he is not less a member of the Association because he may never sit in their meetings. We are not less citizens of the United States because we have not been delegated to Congress; we are citizens of Pennsylvania, contributing to and partaking in its government, though never deputed as legislators. We are Episcopalians, Presbyterians or Methodists, as soon as we are formally admitted into these churches, though we may never be sent to their Conventions.

Do you call this nothing? But all others consider it of great importance to their happiness. See how eagerly the young man of 21 years runs to the polls; see how impatient are foreigners for naturalization, that they too may vote for legislators, when they have no hope of becoming such themselves. The members of County Societies may give their instructions to their delegates; this little privilege is highly valued in politics, why should it not be in medicine?

On the plan now proposed, all power proceeds from the people, that is from the medical public. This accords with our political system; it accords also with our religious system, except in the *Mother Church.* The very essence of this church is to be unchanged and unchangeable, hence it cannot accommodate.

The advantages of our plan are many and more than can be now set forth. The government of individuals would be more perfect. This would devolve upon the County Societies. Each physician, except in large cities, is intimately acquainted with every medical man in his county, and thus all are qualified to judge whether A B or C ought to be admitted or rejected. They are also best qualified to ascertain who is fittest to be sent to the American Association. The County Societies then with open eyes will be the most desirable portals to the medical profession; and their triune diploma will prove a draft for respect and consideration wherever presented.

This organization would annihilate at once all the inveterate jealousy of the schools that now exists in the Profession, by putting all men on a perfect equality in respect to their representative rights. The Professors would be elected Delegates with regard to their talents and learning, and not as at present on account of their fortunate station.

It is of primary importance, to add to the respectability of the County Societies—this would be done by securing the attendance of the *magnates* in the profession, and causing them to take a hearty interest in the business. These Societies are the *Alpha*

and *Omega* of the government of individuals, they are the outposts of the profession, and every means of rendering them respectable ought to be used. Our great men, finding no other portal to the State Society or to the great Association, would attend them more faithfully, and greatly add to their popularity and usefulness.

We should avoid all the confusion and troublesome collisions that now obtain in the election of Delegates. It often happens at present, that the same men are elected from several different institutions of which they are members, as Universities, Hospitals, Colleges, and various societies; now to avoid this unjust accumulation of honors on a few general favorites, much trouble must always be incurred.

The organization proposed would prevent delegations from all unworthy societies. Under the present constitution, as observed above, a few men, two or three, who would not be received into any respectable Society, may suddenly coalesce and send a delegate, with whom you would not be willing to act in a committee.

Dr. Yardley, seeing clearly the many and weighty objections to the present constitution of the Association, brought before our Society, about 12 months ago, a resolution, that it ought to be composed of Delegates from County Societies only. This passed after being considered at three meetings, but with an amendment including delegates from State Societies also. A committee was appointed to lay this resolution before the Association. This was done and a committee of five was then appointed by the Association, and instructed to report at next meeting "whether, to use their own words, some more equitable plan of representation cannot be adopted." To this committee, on motion of Dr. Yardley, the resolution of our Society was referred; so that the subject will be agitated at the meeting in Richmond. To this please to add, that our same resolution was brought before the State Society and almost unanimously approved.

This was a great step towards a reform which might possibly answer nearly all the good ends in view, but it labours under a very troublesome impediment, that of including delegations from State Societies. It is the custom in these to appoint a committee for the purpose of nominating officers and delegates for the coming year—suppose one committee man to be appointed from every county, (and this is the custom) what would he do but recommend for delegate some particular and favored friend? Thus you have a delegate to the Association appointed by one man when he ought to have been appointed by a majority of the whole County Society. There would moreover be some danger from mutual favors among the committee which has got the odious name of log-rolling.

Another trouble would be, that the same man might be elected both by the State and County Societies. It is clear that the avoiding of this would give infinite trouble.

It has been thought by one of our number and perhaps by others, that the Association should be composed of delegates from State Societies only, as more likely to secure a dignified representation.—To this, it may be objected as above, that the members, not having a full knowledge of each other, might often elect those whom they did not know; that each man of the nominating committee would recommend a friend; that mutual favor would introduce the odious log-rolling; that hence a delegate would be elected by a single man instead of a whole County Society.

It would moreover prove very unsatisfactory to a republican people. It is the mode of electing Senators and President, which long since became odious to a majority of our people. This method of electing, to borrow a simile from Swift, is a tag from the aristocratic scarlet coat of John Bull; but on our plain republican garment, it is considered by many as a hideous patch. It must be known to you all, that a bill for changing the constitution in respect to this point, is now before Congress. Whoever would prefer the State Society in the election of Delegates, must entertain the opinion that physicians are not capable of self-government. They must think with an Englishman who once said to me with a sorrowful face—" Oh Doctor, these republican governments were made for men as they ought to be and not for men as they are." Now this comes precisely to the point—physicians approach nearer to what they ought to be than any other people on the earth, and are therefore worthy republicans. Only make the County Societies respectable and they will enforce the edicts of the Association in the most distant regions of America. Of what avail are all these edicts unless there be subordinate authorities to enforce them and carry them to the door and to the bosom of every individual? Congress might make laws every day of their lives, but they would be mere *fulmina bruta* without various organs to execute them.

It has been objected that, by excluding the schools you would shut out many of the most vigorous intellects in the profession. This objection is mere wind or mere words, like Hamlet's letter. Cannot these noble spirits join the County Societies and attend to the duties thereof? The professors are men of business, accustomed to public speaking and debate; their station commands respect; when they come to this floor upon a nominal equality with us, we do not envy them; nay, we are pleased with their condescension, and if they attend faithfully to the business of the Society, we shall prefer them. They have never failed in this house to be elected delegates to the State Society, and they would have been elected also to the Association, but they rather chose to go from their schools.

If it be objected that such an extensive revolution is difficult, I would answer—no. The work would be almost infinitely divided, and among so many that it would be found an easy task. Only let the Association give their fiat and the work will soon be done in all its parts through the whole United States; for it is principally a work of words, in which the American people are said to find great delight. If the Association decree, that in the year 1854, this change shall be made, they will find it done at their bidding. The mere beauty of this universal government would stimulate to the speedy establishment thereof. Who would not rejoice, whose heart would not be gladdened, to see this great empire covered and served by educated physicians, all yielding obedience to one symbol of ethics and to the triune government now proposed.

Gentlemen, it has accorded better with my feeble abilities to give you *hints* rather than *reasonings*, and this you must have perceived in the present discourse; but if you will give your minds to these hints, they will prove *words to the wise* and lead you into further and convincing ratiocinations.

